# Continuity of care for older adults in a Canadian long-term care setting: a qualitative study

**DOI:** 10.1186/s12913-022-08583-1

**Published:** 2022-09-27

**Authors:** Madeline King, Carolyn Steele Gray, Daniel Kobewka, Agnes Grudniewicz

**Affiliations:** 1 Telfer School of Management, University of Ottawa, 55 Laurier Avenue East, Ottawa, ON K1N 6N5 Canada; 2 Bridgepoint Collaboratory for Research and Innovation, Lunenfeld-Tanenbaum Research Institute, Sinai Heath, 1 Bridgepoint Drive, Toronto, ON M4M 2B5 Canada; 3 Institute of Health Policy, Management and Evaluation, Dalla Lana School of Public Health, University of Toronto, 155 College Street, Toronto, ON M5T 3M6 Canada; 4 Clinical Epidemiology Program, Ottawa Hospital Research Institute, 1053 Carling Avenue, Ottawa, ON K1Y 4E9 Canada; 5 Department of Medicine, The Ottawa Hospital and University of Ottawa, 451 Smyth Road, Ottawa, ON K1H 8M5 Canada

**Keywords:** Continuity of care, Goal-oriented care, Long-term care, Older adults, Person-centered care

## Abstract

**Background:**

Continuity of care has been shown to improve health outcomes and increase patient satisfaction. Goal-oriented care, a person-centered approach to care, has the potential to positively impact continuity of care. This study sought to examine how a goal-oriented approach impacts continuity of care in a long-term care setting.

**Methods:**

Using a case study approach, we examined what aspects of goal-oriented care facilitate or inhibit continuity of care from the perspectives of administrators, care providers, and residents in a long-term care centre in Ontario, Canada. Data was collected through documentary evidence and semi-structured interviews.

**Results:**

We analyzed six internal documents (e.g., strategic plan, client information package, staff presentations, evaluation framework, program logic model), and conducted 13 interviews. The findings indicated that the care provided through the goal-oriented approach program had elements that both facilitated and inhibited continuity of care. These factors are outlined according to the three types of continuity, including aspects of the program that influence informational, relational, and management continuity.

**Conclusions:**

Aspects of the goal-oriented care approach that facilitate continuity can be targeted when designing person-centered care approaches. More research is needed on goal-oriented care approaches that have been implemented in other long-term care settings to determine if the factors identified here as influencing continuity are confirmed.

**Supplementary Information:**

The online version contains supplementary material available at 10.1186/s12913-022-08583-1.

## Background

Given the projected rise in the global population of older adults, there is a need for long-term care options that are effective, equitable, and sustainable [1]. As family ties become looser and there are fewer informal caregivers to care for the aging population, community involvement in the care of seniors is becoming more important [2]. In part because of community-based care efforts to help older adults remain independent at home, older adults are entering long-term care settings when they are older, frailer, and in need of more assistance than in the past [2, 3].

Long-term care is “a complex service delivery system comprising a full range of care and support for persons who have, or are at significant risk of having, progressive and/or chronic conditions, and who require services to meet their long-term functional needs” [4]. Long-term care settings face challenges such as staff turnover; lack of staff training; insufficient time allocated to spend with clients; a lack of effective leadership to implement promising practices; privatized and off-site support services; and/or a lack of commitment to ensuring staff, clients, and family members can provide input to improve care approaches [5].

Despite efforts to deliver high-performing long-term care systems around the world [2], many older adults experience a lack of care continuity because of their complex care needs [6]. Older adults are vulnerable to fragmented care since they face barriers to accessing care such as difficulties with memory, reliance on multiple informal caregivers, and difficulties scheduling appointments [7]. These challenges, as well as additional challenges in the long-term care setting, such as staff turnover and insufficient time with clients [5], suggest that an effective model in this setting would focus on reducing fragmentation of care for clients. *Continuity of care* is “the degree to which a series of discrete healthcare events is experienced as coherent and connected and consistent with the clients’ medical needs and personal context” [8]. It has been shown to increase client satisfaction, improve health status, and decrease the use of hospital services [9–11]. A lack of continuity can lead to polypharmacy, medical error and costly overuse of diagnostic tests [12–14]. However, most research on continuity of care focuses on primary care settings, and relatively little work has been done to assess continuity of care in long-term care settings [9].

In 2002, Reid et al. proposed three types of continuity: informational, relational, and management continuity [15]. *Informational continuity* includes the use of information to make decisions for a patient’s care [8], including the use of information about past health events to make informed decisions about current health events [15]. While there is usually more emphasis on documentation of health conditions, information on client values, preferences, and their social context is also important for effective informational continuity. *Relational continuity* is the relationship between the client and the care provider [16]. According to Reid et al., relational continuity acknowledges the importance of “knowledge of the patient as a person,” recognizing that the relationship between a client and a provider is what connects care over time [15]. These ongoing therapeutic relationships bridge discrete health events, for example, allowing the client to know who to contact in the event of a new health problem. Finally, *management continuity* describes the overall management of care, including care planning and the coordination of care [16]. A 2018 systematic review supports continuity of care as a way to achieve desirable outcomes in a long-term care setting [9], but there is little research on how to ensure residents experience continuity of care.

Studies of long-term care emphasize the importance of client-centeredness in achieving high quality care for older adults [9, 17, 18]. A client-centered approach involves staffing that facilitates continuity of care including a team-oriented focus, in-house services, and client documenting in a “resident-centred” way [5]. *Goal-oriented care*, an approach to care that focuses on a client’s individual health goals, is a potential approach to achieving client-centered care [19]. Goal-oriented care involves the client outlining their preferences and care goals, and then the client and care team working together to determine a care plan that aligns with those [20]. For example, if a person living with arthritis and cancer wants to go to church on Sunday, their care plan may include control of pain to perform this activity and social aspects of being able to get to this activity such as getting dressed and arranging transportation.

The goal-oriented care approach, while first identified by Mold in the early 1990s [20], has gained popularity in recent years and is becoming more common in the LTC setting [21]. Goal-oriented care is seen as a “cornerstone to integrated care” as it brings the care team together around the common goals of a patient [22]. This principle and specific characteristics of this approach align with care principles that may influence continuity of care. Specifically, goal-oriented care may reduce fragmentation and improve continuity of care by providing a structured approach to care that focuses on connecting a care team around a client [22]. For example, by recording residents’ goals in a way that is accessible to their whole care team, the transfer of information may be improved, leading to improved informational continuity. As well, residents and family members working with care providers to determine goals of care would likely contribute to a positive patient-provider relationship, leading to improved relational continuity. Finally, a common purpose among care providers to achieve a resident’s goals may facilitate consistency in care, leading to improved management continuity.

We conducted an exploratory case study to provide insight into how care providers, clients, and administrators perceive continuity of care in a single long-term care organization. Our study aimed to determine whether and how a specific goal-oriented care approach influences continuity of care in this setting. If a goal-oriented care approach is perceived to positively impact continuity of care of residents in a long-term care setting, it could be an opportunity for long-term care organizations to improve continuity and quality of care.

## Methods

### Case selection

We performed a case study of the *SeeMe* Program at Perley Health, a long-term care organization. We selected Perley Health, in part, because it is one of the largest long-term care facilities in the province of Ontario, Canada, including seven buildings, eight onsite clinics, and 450 beds [23]. Due to this size, the centre has the capacity for research and innovation, and is known for its innovative approaches to care. The organization prides itself on having the infrastructure, capacity, and expert staff required to innovate, while remaining small enough to “readily implement various models of care” [23].

Upon initial introduction to the centre’s management team, our research team became particularly interested in the centre’s newly introduced approach to coordinating care for clients, the *SeeMe* program. The program was described to us by the CEO as including the elicitation of client goals and the creation of a care plan developed with various providers in a client’s care network. The *SeeMe* program has two components: a comprehensive frailty assessment and a care conference where a resident sets goals in collaboration with their family members and care providers. Our research team recognized that the care conference and the resulting goal-setting components of the *SeeM*e program seemed to align with the basic principles of a goal-oriented care approach. With an interest in the relationship between a goal-oriented care approach and continuity of care, this case was selected to provide insight into a unique approach to care that may have the potential to influence continuity of care in a long-term care setting. The program is further described in the results section based on information collected in the documentary evidence review.

### Approach and rationale

An exploratory case study [24] was conducted including analysis of documentary evidence and information collected through semi-structured interviews. Yin’s case study approach was used as a guide to provide the foundation for understanding when case studies are appropriate, how to develop effective case study research questions, understanding the various types of case studies, and determining what sources of evidence are most effective [24]. Case study methodology was chosen to facilitate an in-depth analysis of the complex relationship between a goal-oriented approach and continuity of care in a real-life long-term care setting [25]. By using multiple sources of data collection, the *SeeMe* program was analyzed to identify themes and develop assertions about the overall meaning derived from the case [26].

### Data sources and collection

Data sources included documentary evidence and semi-structured interviews with administrators, care providers, and residents in Perley Health. Care providers and residents were selected in an effort to capture the perspective of those directly involved in the care experience, and administrators were included to capture broader perspectives on the effectiveness of care approaches in the setting, from an organizational perspective. The documentary evidence review was intended to provide the basis for understanding the *SeeMe* program and its elements and to refine the interview guide to further explore contextual factors influencing continuity. Documents included in the review were the center’s strategic plan, the *SeeMe* information package and care conference agenda, a PowerPoint presentation outlining the rollout of the *SeeMe* program, a PowerPoint Presentation to introduce the program to staff, the program’s evaluation framework, and program logic model. To determine whether a document should be included in the study, the research team assessed whether it was relevant to the *SeeMe* program. Relevancy was determined by deciding whether a document provided contextual information on the *SeeMe* program and/or whether a document provided information to make inferences that could be further explored in the semi-structured interviews [24].

Semi-structured, individual interviews were the primary source of data. Eligible administrators and care providers must have worked for the organization for at least three months, to help ensure that they have a sense of the organizational structure, culture, and how care is provided. Eligible residents were 65 years or older, lived at the centre for at least three months, spoke English, and had been enrolled in the *SeeMe* program. Eligible residents were also required to have a Cognitive Performance Score less than 1 to be included in the study.

Administrative participants were recruited through an email sent by the organization’s research coordinator. Care providers, including physicians, nurses, nurse practitioners, and personal support workers, were recruited using a listserv email sent from the research coordinator. Due to a low response rate (*n* = 2), a second recruitment method was pursued following a snowball sampling technique; asking care providers who had participated to pass on the recruitment email to others who they felt might be interested. To recruit residents, we distributed letters directly to residents by visiting them in their room.

The interviews followed an interview guide (available upon request) and probes were used to elicit more detailed information. The interview guide included questions on participants’ general experiences with the *SeeMe* program, general information about patient care in the *SeeMe* program, and factors affecting continuity of care. There were two versions of the interview guide, one for administrators and care providers, and one for patients. For example, administrators and care providers were asked “what helps achieve continuity of care for clients?,” where clients were asked, “tell me about how well your care providers know you.”

Ethics approval was obtained from the University of Ottawa Ethics Board (S-05–19-2880). Informed consent was obtained from interview participants and all methods were performed in accordance with the relevant guidelines and regulations.

### Data analysis

#### Documentary evidence

Documents provided by the organization for our research were carefully reviewed and analyzed to determine if they were relevant to the research aims, and then summarized in a table organized by rows of what document the information came from. Documents collected in the documentary evidence review were used to provide an in-depth description of the *SeeMe* program and identify factors that could be further explored in the semi-structured interviews. Information was considered “important”, and included in the table, if it was relevant to understanding the context of the program or if it was considered to be an element that related to the research aim that should be further explored in the interviews. Information that was excluded from the documentary evidence, after careful analysis, included information that was not relevant to the SeeMe program or research aims. For example, organizational policies and procedures beyond the SeeMe program. Having an in-depth understanding of the *SeeMe* program in advance of the semi-structured interviews facilitated designing interview questions that provided in-depth information on experiences with the program, rather than using interview time to gain an understanding of the contextual elements of the program itself.

#### Interview data analysis

Interviews were audio recorded and transcribed. Transcripts were imported to NVivo 12, for coding. Thematic analysis was used to identify, organize, describe, and report themes within the data [27]. Due to the nature of the research questions, specifically in terms of the focus on understanding the experience of continuity, an initial codebook was developed using themes from Reid’s conceptual framework including “informational continuity”, “management continuity”, and “relational continuity” [15]. Inductive codes were also developed from the data from the documentary evidence review including “care conference” and “comprehensive frailty assessment”. These codes were contextual elements of the *SeeMe* program that we believed may be brought up by participants in discussing their perceptions of continuity. This codebook was then tested on three interviews (including one from each participant category) independently and in duplicate by the lead author (MK) and senior author (AG. After coding each interview, we met to discuss coding and agree on any additional inductive codes that should be added to the code book until a codebook was finalized. This codebook (see supplementary materials) was then applied to the remaining interviews.

After coding all thirteen interviews, the data within each of the codes and sub-codes was extracted from NVivo as a node report. Node reports were analyzed one by one, pulling out important themes and associated quotes. Relationships between themes were explored iteratively by mapping ideas and combining themes that were similar across codes. Themes that related to perceptions of how the *SeeMe* program or other elements influenced continuity were classified as factors and mapped to determine relationships. Factors were defined as the specific element of the *SeeMe* program influencing continuity. To determine how a factor influenced continuity, they were spilt into facilitators and inhibitors of continuity.

## Results

### Documentary evidence review

The documentary evidence review included six internal documents. Based on this review, we found the *SeeMe* program’s aims were to align care with quality of life goals, incorporating an understanding of what quality of life means for each individual into their care [28]. The program uses the Centre’s frailty-informed care model, involving an iterative process of comprehensive frailty assessments, dialogue to determine the impact of frailty on care, and assessment of how care is aligned with quality-of-life goals. The centre’s frailty informed model is based on the principle that frailty is a strong predictor of health outcomes and understanding and recognizing an individual’s level of frailty is crucial to providing effective care [28]. The first step of the program is a Comprehensive Frailty Assessment that provides information on different drivers of frailty, focusing on major drivers such as cognition, function and mobility [28]. With the results of the assessment, a resident is assigned an overall Clinical Frailty Score. A care conference is then held with the resident and their family to discuss the resident’s overall health and considerations for future decision making. The care conference agenda guides this discussion and includes welcome and introductions, an interdisciplinary care overview, quality of life discussion, medical overview, residents’ values and beliefs discussion, goals of care and future health and personal care preferences discussion, and timelines for follow-up [28]. To guide the goals of care discussion and document the results, a goals of care checklist is used, including options for (1) focus on comfort/symptom management, quality of life; (2) focus on managing illness while maintaining current function/independence; (3) focus on treatment of illness; and, (4) focus on extending life [28]. A future and personal care preference checklist is also used, including options for CPR and defibrillator; transfer to ED for advanced/urgent diagnostics and treatment; stay at Perley Health for diagnostics and treatment; and, stay at Perley Health for: palliative/comfort care, chemotherapy, surgery, medications, dialysis, tube feeding, and ventilator [28].

Treatment options are considered in the context of their frailty as well as their goals, values, and preferences. The care conference and the resulting goal-setting components of the *SeeM*e program align with the basic principles of a goal-oriented care approach. The similarities between the *SeeMe* program and a goal-oriented care approach are outlined in Table 1, using information captured in a review of goal-oriented care literature and information and quotes from the document review.

**Table 1:** Similarities between goal-oriented care approach and *SeeMe* program

Goal-Oriented Care Approach [5, 19, 20]	*SeeMe* Program [28]
Person-centered approach	“The resident and family are at the centre of the model due to the core value of person and family-centred care”
Focus is on client goals across physical and social dimensions	*SeeMe* provides a whole-person approach to understanding residents and their family’s health story
Clients outline preferences and care goals	Care conference agenda includes outlining goals of care
Collaborative process between care team, client, and family	“Program involves true partnership between healthcare team and person/family”
Aim is to achieve maximum quality of life as defined by an individual	Program aims to “align care with quality of life goals, incorporating a true understanding of what quality of life means to an individual”

### Semi-structured interviews

Thirteen individuals participated in interviews including four administrators, four care providers, and five residents. Table 2 presents an overview of the study participants. Interviews were conducted between February 2019 and November 2019. Interviews ranged from 12 to 52 min, with a median interview length of 25 min. Four interviews were conducted over the phone and the other nine were conducted in person, depending on which method of communication was most convenient for the participant.

**Table 2:** Overview of study participants

Participant Type	n
Administrators	4
Care Providers	4
Physician	1
Nurse	2
Registered Practical Nurse	1
Residents	5

Results of the thematic analysis showed that the *SeeMe* program was perceived to both facilitate and inhibit continuity of care for residents at Perley Health. See Table 3 for an overview of these results, organized by type of continuity.

**Table 3:** Aspects of the goal-oriented approach perceived to be facilitating and inhibiting continuity

	**Facilitators**	**Inhibitors**
Informational Continuity	• Goals of care discussions ensured resident, care team, and family were on the same page • Care conferences created awareness for residents of their care options • Consistency in where *SeeMe* assessment information is stored	• Residents lacked awareness of *SeeMe* program • Care conference agenda caused confusion
Relational Continuity	• Incorporating a resident’s values and preferences formed a holistic understanding of a resident • Staff increased awareness of the program for families • Integrating the family’s perspective into a resident’s care	• Relying on family involvement when family was not available
Management Continuity	• *SeeMe* program discussions facilitated informed care preference decisions • *SeeMe* assessments acted as reference tool in case of acute health event • Goals of care discussions empowered residents and family members to talk to external healthcare providers • Structure of the *SeeMe* program facilitated consistency in care being provided	• Wait time until residents attended their first care conference was too long • Family members faced difficulties making decisions

#### Informational continuity

Informational continuity includes the use of information to make decisions for a patient’s care [8]. This involves the use of information about past health events to make informed decisions about current health events [15].

#### Informational continuity facilitators

Administrators and care providers felt that the *SeeMe* program goals of care discussions ensured the resident, care team and family were on the same page*.* These participants felt that if a family member and a resident did not initially agree on what goals were most important, the goals of care discussion helped form a mutual understanding of what was really important to the resident. Specifically, by understanding the context around why certain goals are important to a resident, everyone involved in a resident’s care had a foundation on which to base their decisions.

Administrators and care providers also felt that the care conference created awareness for residents of their care options. Through the care conference discussion, care providers could educate residents and family members about the care options available to them. Care conferences provided families with information on options that were potentially more comfortable and less invasive that avoided transferring residents to different healthcare settings, such as hospitals:

*I think it’s also highlighted what long-term care homes can support in our environment. So the perception of what we have the ability to do from a medical perspective, IV therapy and PICC lines, and we manage pain very nicely, and we can do these things on site, is not something that the general public knows... And having those conversation during the SeeMe discussion has been another very good educational component for families* – Administrator 3

Administrators and care providers noted that, with the *SeeMe* approach to care, there was consistency in where *SeeMe* assessment information was stored. Information from the comprehensive frailty assessment and care preferences and goals from the care conference were recorded in a resident’s EMR so all care providers could refer to this information at any time. Care providers emphasized the importance of easily accessing resident information when they were covering shifts on units where they do not know the residents. All care providers having easy access to the same information ensured the care they provided was based on a similar understanding of the resident, as demonstrated below:

*I have a high risk guy to see after I talk to you today. And that goals of care and future healthcare tool will help inform me where we’re going to really...where I’m going to kind of centre my discussion. I’m not going to go down and have a discussion about like transferring to acute care. I’m going to go down, read their notes, understand their values, understand their understanding of illness and trajectory, and then help support them in that conversation.* – Care provider 1

#### Informational continuity inhibitors

Residents lacked awareness of the program. Only one resident was aware of the goal-setting component of the program and no residents recognized the name of the *SeeMe* program itself. Most residents interviewed felt they had not been asked about their goals of care or care preferences. Since residents were not aware of conveying this information to care providers, perhaps the way in which goals were elicited was not framed as a “goal setting” activity from the perspective of residents, and/or the information that was recorded for a resident’s goals and care preferences was not an up-to-date reflection of their values and needs. Despite this, residents seemed satisfied with their care.

*Interviewer: And have you ever talked to any of your care providers or anyone here about your goals of care, what you want for your care?*

*Participant: No, I’ve never raised any questions because the care I’m getting is quite satisfactory.* - Resident 1

Care providers also expressed that the care conference agenda caused confusion, noting that it often brought up conversations that would otherwise not be raised with the resident and their family. These were thought to be longer and more overwhelming conversations than were necessary. Specifically, since the conference agenda was standardized and comprehensive, it resulted in discussions that were not always relevant to the resident.

*For very frail patient with dementia in her 90s, I would not broach the topic of chemotherapy or dialysis in my care conference, you know, anyway. So now, you know, because there’s that questionnaire in the SeeMe program, I ask for it. But sometimes it opens or the potential to open some pandora’s box with certain families that I wouldn’t have necessarily opened before the SeeMe Program.* – Care provider 3

#### Relational continuity

Relational continuity is the relationship between the client and the care provider [16]. This relationship involves not only bridging discrete events in the past, but also providing a link to future care.

#### Relational continuity facilitators

Administrators and care providers felt that the *SeeMe* program involved incorporating a resident’s values and preferences to form a holistic understanding of a resident. Health information, such as current medications, health and family history, as well as personal information such as family, previous career, interests, and hobbies, were shared during the care conference discussion. Administrators and care providers felt this helped the care team and resident form a relationship that was more personal than caregiver-provider relationships in the past.

*We have just heard some really very heartwarming stories of staff who have really bonded with the residents and how they have really got to know them, know their past life stories. I know there are several PSWs who sing, they know all the residents’ favorite songs, maybe they will sing to them in the shower to decrease their anxiety. There’s just, you can see that there’s this bond established between them...and I think that makes such a huge difference -*Administrator 2

Administrators and care providers explained that staff increased awareness of the program for families by meeting with the family and resident before the resident moved into the Centre. In this initial session with the resident and family, the care provider described the program and how it influences the approach to care at the Centre. Residents and their families also completed paperwork so that on their day of admission the focus could be on building relationships with their care providers rather than administrative tasks. Administrators and care providers felt these conversations between staff, family members, and residents built trust as family members could learn about the approach that was guiding the care of their loved one.

*So now we’re having these trusting, open, honest conversations. So we’ve got trust with residents, trust with families. When we say we’re going to be there for their crisis, we are there for their crisis. We’re stepping up to the plate to show them we heard you, we listened, and we responded.* - Care provider 1

Integrating the family’s perspective into a resident’s care through the *SeeMe* goals of care discussion facilitated relational continuity. Administrators and care providers found that through the care conference, family members provided information on what a “normal day” or “normal behaviours” for a resident looked like before they moved to the Centre. This information can often only be provided by family members when residents have difficulty remembering their previous life. Using the family’s unique perspective and integrating this information into a resident’s care brought the resident’s care team together to focus on the specific needs of a resident and made their quality of life as high as possible.

*To say to a family, “when things were good what did a normal day look like?” And let’s try to make this as normal as we can. And if normal was always watching the midnight news then that’s okay. And not to be structured by the clock and the task but to focus on the resident and the quality of life for him or her. That movement to the team approach and that everybody is here to support the resident.* – Administrator 3

#### Relational continuity inhibitor

Care providers expressed that the program relies on family involvement but family was not always available. Family involvement, required for care conferences and ongoing conversations to ensure care was aligned with values and preferences, created challenges when family members needed to make decisions on the behalf of their loved one but were not available. This challenge hindered the ongoing relationship between care providers and family members by creating an obstacle for effective collaboration.

*So especially for residents who have dementia and cannot give consent about sharing information, so we rely on power of attorney or substitute decision-makers. Usually it’s a family member. And one of the challenges is some families don’t have necessarily the same opinion on the care. So usually we have a point of contact person to call first. But if we cannot join that person, we’ll call the next one in line, and so and so forth.* – Care provider 3

#### Management continuity

Management continuity describes the overall management of care, including care planning and the coordination of care [16].

#### Management continuity facilitators

Care providers and administrators felt the SeeMe program discussions facilitated informed decisions. Specifically, the frailty assessment was perceived as helping the care team and family make decisions based on the resident’s frailty level.

*We put it all into an assessment and from that we can get a specific level of frailty. With that level of frailty, the doctor and the team can have a better idea of what to expect or how vulnerable that person is to healthcare changes. With that number [frailty assessment score] and that understanding of their frailty, we bring it to the care conference and talk to the family about what that level of frailty means. We ask questions about what they want to see in their life here for quality and then together we can make healthcare decisions.* - Administrator 1

Administrators and care providers consistently mentioned that the *SeeMe* assessments acted as a reference tool in the case of an acute health event. When an acute health event occurred, for example a resident fell and broke their hip, the family became involved in a discussion with care providers where they decided whether to change their care goals or keep them the same. Providers and administrators felt that by having goals of care discussions in advance, residents and family members were already informed of the risks and benefits of different care paths, and this simplified the decision-making process.

*Because let’s say an event were to happen, the intent is for the staff or physicians to touch base with the family and say, “when we last discussed this you said this...now that this event happened is this still where you want to go?” and here is a review of the risks and benefits of each path. If you choose path B then here’s the benefits...It is meant to inform discussion, but not have it be set in stone”* – Administrator 3

Administrators felt the goals of care discussions empowered residents and family members to talk to external healthcare providers*.* They described that by establishing care preferences and goals through the *SeeMe* program, residents and their family members had a clear understanding of what they wanted from their care and were able to convey this information to their healthcare providers. Administrators perceived that residents and family members also recognized the impact of having a voice in their care on the resident’s quality of life. Empowering residents and their family members to discuss their care preferences and goals with external healthcare providers facilitated a more consistent care experience across healthcare settings.

*I know two instances since the SeeMe program […]Where a family has felt empowered to talk to external healthcare providers, around the goals of care of their loved one. Because they felt informed, they understood the risks, they know frailty, and they understood frailty and its response to care delivery. And felt very supported in decisions that they were making because they knew that the home themselves, if there were questions, would be able to support that.* – Administrator 3

Administrators and care providers felt that the structure of the *SeeMe* program facilitated consistency in care. Specifically, the care conference structure ensured that all residents and their families set goals in the same way and had similar discussions about how they prefer to receive care. As a result, it was perceived that care was provided in a similar manner across the Centre.

*... we’re all kind of on the same page. So we all understand frailty, we understand trajectory, we understand terminal illness, we understand those drivers of frailty. So for me not only is it continuity around language care, you know, professionals, but that like we’re all speaking...we’re all doing kind of the same thing.* - Care provider 1

#### Management continuity inhibitors

Care providers noted that the wait time until residents attend their first care conference was too long*.* One care provider shared that the first care conference occurs six weeks after admission. The participant noted that some residents do not make it to the six-week care conference before transferring to another care setting or passing away. As a result, these residents were not able to express their goals and care preferences to guide their care. Care providers echoed that decisions about goals and care preferences may be especially important for people near the end of their life, as they may choose very different care paths depending on their goals during this time.

*They’re done within the first 6 weeks of the care conference. And then annually after that. I think one of the challenges we’re facing is those residents who come in who are imminently dying. Like they don’t make it to the 6-week care conference. Those guys, we’ve got to figure out a way to capture those guys a little bit sooner.* – Care provider 1

Care providers felt that family members faced difficulties making decisions during the care conference. While the end result of care conferences usually meant highly informed and well-thought-out decisions, the process of family members making these decisions was often difficult, specifically when they were encouraged to consider how these decisions aligned with the resident’s values, goals and preferences. Since the care conference agenda included all aspects of care, including end-of life care decisions, families were sometimes offended and often not ready to make such difficult decisions about care that they did not see as imminent.

*It’s a hard topic for people. I’ve seen people come into the care conference—family and that—and they have a really difficult time talking about end of life for their parent. It’s like something that they haven’t faced yet.* – Care provider 4

## Discussion

This qualitative case study indicated that the care provided through the *SeeMe* program had elements that were perceived to both facilitate and inhibit continuity of care as defined by Reid’s framework. The *SeeMe* program is a unique approach involving a frailty assessment to determine a resident’s overall level of frailty, followed by a care conference involving a resident, their family, and care providers where participants discuss quality of life, medical status, residents’ values and beliefs, goals of care, and future health and personal care preferences. Our findings demonstrated that the *SeeMe* program aligned with the basic characteristics of a goal-oriented approach, including a consultation between healthcare providers and patients to determine a resident’s goals and create a care plan. A common theme across the three aspects of continuity was that the *SeeMe* program brought the care team together to work in a coordinated effort with the resident and family.

The *SeeMe* approach to care is similar to the goal-oriented approaches evaluated in recent studies by Blom and colleagues’ study on Integrated Systematic Care for Older People [29], and Steele Gray and colleagues’ study on the usability and feasibility of an electronic tool designed to support goal- oriented primary care delivery [30]. The positive relationship between a goal-oriented care approach and continuity of care, as perceived by participants in this study, aligns with the outcomes of these two key studies. Specifically, both Blom and Steele Gray found that care planning using a goal-oriented approach created stability in care and ensured everyone involved was on the same page [29, 30]. The ability of this care approach to bring a care team and patient/resident together is especially important in fragmented care settings, where care approaches such as goal-oriented care can help bridge the gaps between those providing and receiving care.

Although the *SeeMe* program attempted to base care around resident’s goals, values and preferences, this approach had the potential to increase delays in goal-oriented care (due to a long wait time until the first care conference) and confusion among residents and their family members (when discussing care pathways not relevant to the resident), negatively influencing all three types of continuity. Steele Gray et al. similarly found that when providers used an electronic tool for implementing goal-oriented care, monitoring questions did not always address individual patient needs, did not always fit well with provider workflow, and made daily reporting more time-consuming [30]. With these potential pitfalls of a goal-oriented care approach identified in the primary care setting, it is likely that these challenges will be magnified in the long-term setting under stressful conditions working with frail individuals and with complex care needs, as our study showed. Hence, it is important that goal-oriented care approaches are designed to align with the workflow in the organization and ensure simplicity for providers and patients and residents where possible.

This paper also provides insight into specific aspects of care that are related to continuity in the long-term care setting. A common theme across all three types of continuity was that the *SeeMe* program was perceived to positively influence how consistent care was for clients, including consistency in where information was stored and in the care experienced by residents. Similarly, one of the few studies focusing on continuity of care in the long-term care setting, assessing the Care by Design program in Halifax, Nova Scotia, found that continuity of care was improved when there were tools in place to create consistencies in medical care such as care directives and notes in charts [17]. These findings suggest that the basic approach of having a care model that includes explicit standards of care may have a greater impact on continuity than the specific components of a goal-oriented care approach. Regardless, since we know the importance of client-centeredness in achieving high-quality care for older adults in long-term care settings [31], perhaps goal-oriented approaches can offer a client-centered approach that improves continuity. Further research is needed to explore the relationships between goal-oriented care and the three elements of care continuity, specifically, further identifying what mechanisms of goal-oriented care enable continuity of care in this setting.

This study has some limitations. Firstly, it is a single qualitative case study of a unique program, limiting the transferability of the results to other long-term care settings. Second, we were limited in our ability to recruit physicians, and the individuals participating in the study were likely to have an interest in the *SeeMe* program and thus not be reflective of the opinions of other stakeholders in the Centre. Since we interviewed a limited sample of administrations, care providers, and clients, we were limited in the perspectives that we captured and may have not included participants that could offer other insight into the program and how it influenced care. For example, family members may have offered a unique perspective that was not captured in our work. Our ability to engage with residents during the semi-structured interviews was hindered by their lack of awareness of the program. Future work would benefit from including family and informal caregivers in the interview who may be more familiar with the programs offered. Lastly, our definition of goal-oriented care focused on health goals, which may have excluded life goals beyond the medical domain. Despite these limitations, we were able to capture unique insight from care providers, administrators, and residents on their experience with a goal-oriented care program in a Canadian long-term care setting. The results may guide improvements in care by leveraging specific elements of a goal-oriented approach, such as the care conference model or the frailty component as a starting point for further development.

## Conclusion

Initiatives such as the *SeeMe* program that incorporate person-centered and goal-oriented care may help facilitate continuity of care for older adults in a long-term care setting. When designing care approaches, aspects of the *SeeMe* program, such as care conferences and standardized practices for what information about a client is collected and how that information is stored that facilitate continuity can be targeted. The inhibitors of continuity identified in this research should be noted by others when designing care approaches to achieve continuity. Future longitudinal research could examine the specific outcomes of improved continuity of care for long-term care residents in facilities that have implemented a goal-oriented approach. In these times of witnessing the impact of the COVID-19 pandemic on older adults in long-term care, it is important to improve the quality of life and care in long-term care. Goal-oriented care approaches have the potential to ensure that care is focused on residents’ unique needs and wishes, offering a care approach that may improve lives and outcomes.

## Supplementary Information

**Additional file 1.** Codebook.

## Data Availability

The datasets generated and analysed during the current study are not publicly available to protect the confidentiality of the participants. Qualitative interview transcripts could be used to identify participants and cannot be shared publicly as per research ethics board guidelines.
