# Supplementary material for: Continuity of care for older adults in a Canadian long-term care setting: a qualitative study
Source: BMC Health Serv Res. 2022 Sep 27;22:1204. doi: 10.1186/s12913-022-08583-1 (PMC9512963; doi:10.1186/s12913-022-08583-1)
Supplement: Supplementary file 1 — Additional file 1. Codebook. [file 12913_2022_8583_MOESM1_ESM.docx]

## Supplementary Material

## CODE BOOK

| **GRANDPARENT CODE** | **PARENT CODE** | **CHILD CODE** |
| --- | --- | --- |
| **Perley & Rideau:** describes information regarding the Perley & Rideau at multiple levels including organizational, staff or resident | **Organizational information:** describes information regarding the Perley and Rideau in general |  |
|  | **Staff Information:** describes information on staff including care providers and administration |  |
|  | **Resident information:** describes information on residents living at the Perley & Rideau | **Resident experience:** describes the experiences of residents living at the Perley & Rideau |
| ***SeeMe* Program:** Perley & Rideau’s frailty-informed care model involving a frailty assessment followed by a care conference to determine a residents care goals | **Organizational Level:** describes how the *SeeMe* program influences elements of the Perley & Rideau’s organizational structure, culture, and overall function | **Philosophy of Care:** values, beliefs and ethics regarding care and treatment of residents in the SeeMe program |
|  |  | **Implementation:** describes how the program was implemented throughout the organization |
|  |  | **Organizational outcomes:** describes outcomes of the SeeMe program that effect the organizations structure, culture and overall function |
|  |  | **Leadership Buy-in:** support and agreement from senior leaders on the mission, vision and values of the *SeeMe* program |
|  | **Staff Level:** describes how the *SeeMe* program influences staff and how staff influence the effectiveness of the *SeeMe* program | **Education & training:** Used to describe education and training efforts that are made to teach care providers about the *SeeMe* program and how they should be using it in their work. |
|  |  | **Roles:** Describes what care provider’s role in providing care through the SeeMe program is. |
|  |  | **Staff Buy-in:** support and agreement from care providers on the mission, vision and values of the *SeeMe* program |
|  |  | **Staff level outcomes:** describes outcomes of the *SeeMe* program that affect staff at the Perley & Rideau |
|  | **Resident & Family Level:** describes how the *SeeMe* program influences residents and family members and how residents and family members influence the effectiveness of the *SeeMe* program | **Program awareness:** describes how aware patients and family members are of the SeeMe program and its care components (e.g. frailty assessment, care conferences, and resulting care as a result of these assessments) |
|  |  | **Autonomy:** Freedom to make ones own choices. Use this code to describe information about patient having more or less control over their decisions. |
|  |  | **Patient level outcomes:** describes outcomes of the *SeeMe* program that effect patients and families |
|  | **Approach to care:** describes the care that is provided through the *SeeMe* program | **Holistic:** to describe care that is based on an understanding of the client as a whole person, not just a patient or diagnosis. Includes an understanding of their physical, psychological, emotional and spiritual dimensions. |
|  |  | **Structured approach:** describes an approach to care that has clear procedures and protocols |
|  |  | **Person-centered:** to describe care that is focused on the patient and their preferences, values and goals. Apply this code for any reference made to this type of care. |
|  |  | **Frailty informed care:** looks at health decisions that would best suit a frail adult. Use this code for any action or decision that is frailty-informed. |
|  |  | **Goal-oriented care:** approach to care that focuses on a client’s individual goals |
|  |  | **Family involvement:** Describes health events or decisions that involve the resident’s family. |
|  |  | **Non-holistic/non-structured/non-person centered/ non-goal oriented care approach:** describes care that does not follow the principles of either holistic, structured, person-centered or goal oriented care. |
|  | **Comprehensive Frailty Assessment:** assessment conducted by a care provider on a resident that assigns a frailty score based on information on different drivers of frailty | **Frailty Score:** Clinical score assigned to a resident based on an assessment of a residents cognition, function and mobility. Scale ranges from 1 (very fit) to 9 (terminally ill). |
|  |  | **Frailty drivers:** Major drivers including as cognition, function and mobility that effect a person’s frailty |
|  | **Care Conference:** meeting held with the client and their family to discuss the overall health picture and considerations for future decision making. During this meeting clients are asked to discuss their goals, values and preferences with the care team. | **Goal setting:** Creating goals after frailty-assessment to be used to treat health conditions. Describes choices that the resident gets to make about their health care plan and health outcomes. |
|  |  | **Care Plan:** Describes the care plan that is a result of the goal-setting process. Includes information on how a resident would like to be cared for daily as well as in the case of an acute health event. |
| **Informational Continuity:** includes the use of information to make appropriate decisions for a client | **Information** **Transfer**: the transfer of documented or verbal information from one provider to another | **EMR:** Electronic Medical record. Apply code for any reference to processes or tools on this system. |
|  |  | **History:** Information on a patient’s past including personal and health information. |
|  |  | **Assessment & Report:** Used to describe assessments conducted by care providers to record information about a patient and reports to inform care providers of important information (e.g. shift report) |
|  |  | **Discussion:** Used to describe any face-to-face information sharing between staff or residents and staff. |
|  | **Provider knowledge:** extent to which care provider is aware of information about a resident | **Provider knowledge of resident health:** the extent to which a provider is aware of health status information about a resident |
|  |  | **Provider knowledge/use of resident care plan:** degree to which a provider is aware of a residents care plan and the extent to which they refer to the care plan to provide care |
|  | **Patient & family knowledge:** extent to which a resident is aware of their health status and care plan. Use this code with health status and care plan information overlap. | **Patient & family knowledge of health status:** Describes the information that a patient knows about their health status including level of frailty, comorbidities, cognition, function and mobility. |
|  |  | **Patient & family knowledge of care plan:** Describes the information that a patient knows about their *SeeMe* care plan and their associated goals of care |
| **Relational**  **Continuity:** the relationship between the client and the care provider | **Client-provider relationship:** describes the relationship between clients and their care providers (PSWs, nurses, physicians, and any informal care providers). Use this code when care information and personal information overlap. | **Care relationship:** Used to describe relationships between care providers and residents. Refers to how well a care provider knows a resident’s care preferences, frailty, and health status. |
|  |  | **Personal relationship:** Used to describe relationships between care providers and residents. Refers to how well a care provider knows a resident’s history, family, hobbies, interests etc. |
|  | **Consistency of care providers:** describes the variability in care providers providing care to a client | **Short-staffed:** Indicates any reference to a shortage of staff in the organization, causing difficulties providing effective care. |
|  |  | **Staff rotation:** When care providers have to rotate their shifts to be with different residents, meaning they are not always with the same resident. |
| **Management Continuity:** describes the overall management of care, including care planning and the coordination of care | **Consistency of care:** describes the variability in the experience of care for a client from shift to shift or day to day | **Process & procedure consistency:** Used to describe process and procedures that create a consistent resident experience across the organization. |
|  |  | **Teamwork & Coordination:** Describes any activity that involves care providers working together to provide care to residents and/or family members working with care providers to provide care. Also use this code when multi-disciplinary teams are discussed. |
|  |  | **Transfer across care settings:** the extent to which the care experience remains consistent across care settings (e.g. long-term care to hospital) and the extent to which care providers are aware of care needs and preferences across care settings. |
|  | **Flexibility of care:** the extent to which care is individualized to a resident and the actions of adjusting care to meet the needs of a resident in a specific instance | **Dynamic:** Used to describe a process that involves constant change and adaptation as a residents care needs and preferences change |
|  |  | **Acute health event:** Used to describe any sudden change in a patient’s health conditions. Use this code when describing crises or reponse to acute events. |
